# Medicine in North America

**Published:** 1963-10

**Authors:** John Apley


### MEDICINE IN NORTH AMERICA

BY

JOHN APLEY, M.D., F.R.C.P.

"Intellectual eunuchs who do not propagate their own kind" is a characteristically vivid description that was applied in the United States of America to professors whose duties do not include teaching. There was no danger of my being included in this category when I went to Louisville University as visiting professor in paediatrics, for teaching was my main task. During the three months of my stay I was a member of a very active department; but because, as a visitor, I was spared administrative chores, there was time to look and listen, to ask questions and discuss problems, to to learn as well as teach. Time was made also for me to accept invitations to lecture, take rounds, and participate in discussions at other hospitals and universities, and I travelled 12,000 miles within the U.S.A., from Colorado in the west to Philadelphia, Yale and Harvard in the east. When I was working in Boston in 1948–49 I certainly could not have predicted the developments in medicine that were to occur within a decade or so in a country so enterprising and energetic as North America.

Bristol and Louisville are cities with roughly the same population; how do their medical schools compare? The medical school in Louisville, founded in 1837, is larger than its Bristol counterpart, admitting 100 students annually. The most distinguished name attached to the school is that of Austin Flint, at one time Professor of Medicine. The proportion of general practitioners produced is high for an American medical school. The amount of research is considerable by English standards and a magnificent research institute has just been completed on the medical campus.

As regards paediatrics, apart from an international growth study centre, which is incorporated in the paediatric department, the training facilities are similar to those in Bristol. The main children's hospital seemed reasonably modern to English eyes, but not to my colleagues, for it is 13 years old. Its 150 beds admit private patients under the local paediatricians, though these patients are available for teaching; in addition there are some 30 beds for children in wards of the general teaching hospital nearby, together with large departments for newborn and premature infants. The clinical material is extremely varied and apparently inexhaustible; I saw all the disorders we see here, more sickle cell disease and surprisingly more malnutrition.

In the remarks which follow I shall attempt to sketch a picture of medical training and practice that is not restricted to Louisville or to paediatrics.

### MEDICAL STUDENTS IN THE U.S.A.

I quote some strong words from an essay by an English medical student (Colt ,1962) from University College Hospital who spent two months at Duke University, North Carolina:

"The ward work was a rude awakening from the leisurely pace of an English Medical School. After the first week, I felt so tired, antagonized and inadequate that I wanted to go straight back to London.

The students had to be on the wards at 7.30 a.m. There was a two hour teaching round starting at 10.00 a.m., six days a week. Every patient admitted had to be presented on the following day on this round—even if he was admitted in the small hours. I put up an I.V. drip and did a lumbar puncture for the first time. We also had to do nearly all the laboratory work on the patients except the Biochemistry.

That is, Haematocrit, Haemoglobin, Peripheral Smear, W.B.C. and Differential, urinalysis, stool examination, etc.—and even L.E. cell tests when necessary! History writing took up a vast amount of time as *seven sides of quarto* were expected as an average. There were conferences on Radiology, Cardiology, Neurology and Haematology each week and a Clinico-Pathological conference at noon each Saturday, as well as the Intern's round six days a week from 9.00 a.m. to 10.00 a.m.

After finishing on the wards, which was rarely before midnight, we used to look through the Journals for a couple of hours reading about topics related to the patients. I learned more in the two months of the clerkship than ever before or since."

I have tried to estimate the little time that this industrious student seems to have had for sleeping; but I will not even guess at what the sister in charge in an English hospital would say if he appeared in her ward at 7.30 a.m.

That American students work harder than English students is a common generalization which seems justifiable. They are older when they start their medical studies, many of them are supported financially by loans (or by a wife who goes out to work) and they have only two years in the wards before internship. They are not so bedevilled by examinations. They read more and argue better than English students. They find less time to spend with patients and to cultivate clinical skills.

### HOSPITAL MEDICAL STAFF

In proportion to the number of patients there are twice as many junior staff in American hospitals as in English hospitals. Throughout the U.S.A. their number has increased by about a half in the last 16 years, but they still do not meet the increasing requirements of sophisticated modern medicine. Perhaps a fifth of the junior posts are filled by foreign graduates, who tend to be appointed to hospitals where opportunities for learning are not of the highest standards (Bowers, 1963).

It seems illogical to non-Americans that so many decisions regarding patients are made by the junior staff, rather than by seniors of much wider experience.

Since the war the number of doctors seeking training in a specialty has increased six times or more. This vast expansion has been controlled to some extent by 19 specialty boards which are establishing standards for certification. Graduates who intend to specialize follow a required course of study in their chosen subject for at least five years before they can satisfy the board requirements. It seemed to me that most are too soon and too narrowly confined within their specialty.

Most departments in teaching hospitals employ Research Fellows, and indeed the status of the department or medical school may be too readily equated with the number of financial grants it obtains and the number of Research Fellows it attracts.

In many hospitals board specialists, like the 50 paediatricians who are primarily engaged in private practice in Louisville, are in turn selected for periods to do rounds on in-patients. They may also be responsible for much of the out-patient work, and in teaching hospitals this often includes supervision of medical students who take the histories and examine the patients. There are hustling, bustling general clinics that resemble our casualty departments; standing out in contrast are the many special out-patient clinics in which experts provide a truly personal service that ensures continuity and is often of outstanding excellence.

At the head of a teaching hospital department, such as medicine or paediatrics, is the Chairman, usually a full-time Professor, who is largely responsible for organisation, administration and finance. He usually undertakes in addition some clinical work, teaching and research, though much of these is delegated to other full-time or part-time Professors in the same department, or to Associate or Assistant Professors, who are sometimes engaged also in private practice. The increasing administrative responsibilities of heads of teaching departments in America (as in other countries) tend to encroach more and more on the time and energy that they can give to clinical medicine.

### CONSULTANT AND PRIVATE PRACTICE

In proportion to the population there are rather more doctors in the U.S.A. than in Britain, but one hears in most places of an increasing shortage. An outstanding difference is the proportion of specialists to general practitioners; for every three general practitioners in Britain there is one specialist, while in the U.S.A. there are no fewer than five specialists. Nevertheless, in America there are signs of a resurgence of interest in family doctoring. Family care is being deliberately studied and taught in some medical schools, both to undergraduates and graduates, and in a few schools the teaching of "comprehensive care" is the subject of interesting experiments.

In Britain there are about 250 paediatricians, consultants based almost exclusively on hospitals and seeing patients referred by family doctors. In Britain, 3 per cent of all specialists are paediatricians; the corresponding figure in America is 8 per cent. In the U.S.A. there are about 12,000 paediatricians, a number twelve times higher than ours in proportion to the population. But most of them are family or general practitioner paediatricians, and much of their work is concerned with minor ailments, prevention and advice. In Britain the "G.P. specialist" is almost extinct, but in America many general practitioners do operative surgery, for example, and the levels of skill and experience inevitably are much more variable than here.

An important difference between the two countries is seen in their systems of "closed" and "open" hospitals. In Britain most hospital beds are in the charge of consultants appointed to the staff. In the U.S.A. the practitioner may continue to attend his patients in hospital, with the exception of university departments and "services" for those patients without a family doctor; but even in these units selected practitioners may take a share in the medical work and in teaching. "Tissue committees", in which the quality of medical care and the results of treatment are examined, assessed and criticized by colleagues, are an admirable feature of some American hospitals which we might copy with advantage.

A semi-retired doctor told me ruefully that "The telephone has now become the most used diagnostic instrument", and American practitioners undoubtedly do give a great deal of advice by telephone. Some doctors go to see only a very small proportion of patients in their homes, a much criticized state of affairs which is apparently not due to pressure of work alone. It is encouraged by the bias of medical insurance towards hospital treatment, by an ingrained belief that investigations are indispensable in almost every case, and perhaps slightly by the fact that the average American practitioner enjoys better accommodation and has more modern equipment than his British counterpart. His expenses are higher, but on the whole he probably earns more in relation to the cost of living.

As regards the finance and structure of medicine, I believe that nobody should assume that a system satisfactory for one country will be equally satisfactory for another. While they have no National Health Service, a considerable proportion of the Americans contribute to societies that provide partial financial cover for medical expenses; but medical care can be extremely, even ruinously, expensive, and to a visitor it is unexpected to find even "service" patients in hospital being charged 75 cents an hour for oxygen and 25 dollars for each consultation with a hospital specialist. On the other hand, generous voluntary efforts, like the "Community Chest", provide vast sums for hospital building and equipment and stimulate an admirable liaison between the community and its hospital services.

### SOME COMPARISONS

If, as Oliver Wendell Holmes wrote, "The chief end of man is to frame general propositions, and no general proposition is worth a damn" I can safely indulge in some general propositions and on them base comparisons between American and British medicine.

Individual patient care, both in domiciliary and in hospital practice, seems from what I saw to be on a more personal and higher level in our country. We owe this, in part at least, to the unaltered predominance of general practitioners outside hospitals, and to the continuing tradition of individual responsibility of the senior staff for hospital patients.

I deplore the gulf, so much more obvious in our country, between practice outside and inside hospital. I should welcome an increase to American levels in the numbers of our hospital staff, but would prefer that our hospital doctors continue to spend most of their time in wards and clinics rather than in laboratories, as in America. In this country the N.H.S. has abolished the problem of whether the patient can afford consultations and treatments, though I suspect that under our system patients are more likely to be kept in hospital longer than is essential or desirable.

Signs of a disuse atrophy of clinical skills and judgment can be seen in Britain, but to me they seemed much more apparent in America. Some of the finest clinicians I have met anywhere are Americans; but on the whole in the U.S.A. there is a widespread tendency to over-reliance on investigations, and a cynic there has remarked that the M.D. degree denotes "Mechano-Diagnostician". I think the erosion of clinical medicine starts at the student level, for two years in the wards before internship is hardly sufficient time in which to develop clinical confidence, but it continues at all levels.

The co-ordination of undergraduate studies has advanced further in America; on the other hand our students are fortunate in spending more time with patients, and have better opportunities of watching experienced doctors handling a case from start to finish.

When I pressed an American student for details of the clinical examination he rebuked me politely by remarking "We can afford the laboratories and apparatus, so why not use them?" But we should not be smug, and I wonder if post-war medicine in Britain might not have gone further than it has along this same path if more money had been available here? Most of us here prefer the traditional attitude that the clinician, not the technician, must make the final decision; but we must guard against becoming clinical Canutes, trying to hold back the tide of scientific progress.

Medical research in America is on a vastly greater scale than in Britain, and the generous financial help available makes our provisions appear niggardly and half a century out of date. But it is unfortunate that in America, much more than here, the kudos attached to research, and the opportunities it affords for advancement, draw potentially good clinicians increasingly from clinical practice and teaching, to which many are better suited and which cannot do without them. As I see it, the correct balance for medicine as a whole has not been struck in either country.

The administration of American hospitals seemed to me to allow for more flexibility than our own as regards equipment, staffing and new developments of all sorts. Indeed, American medicine, as a whole and in its various branches, gives the impression of being readier than its British counterpart to probe and examine its approach and its methods—and to act promptly and vigorously on the results. Almost all the remarks I have made here were voiced by American colleagues.

### POSTLUDE

Even among nations with the same language and tradition the evolution of medicine does not proceed at the same pace or along identical paths. The dissimilarities may be either fortuitous or a result of adaptation; developments that may be appropriate in one social climate may or may not be desirable in another.

At the present time many trends in American medicine are being widely followed in other countries. It is prudent to examine them, to decide where we can benefit from American enterprise and vigour. British contributions to medicine have been outstanding, and in many respects we will rightly prefer to continue to blaze our own trail; but in this period of unprecedented development it is more than ever essential to learn from the experiences of other countries.

Reading about them is not enough. Paying a short visit abroad is to graze only in selected pastures. The best and surely the most enjoyable way to share the experiences of foreign colleagues is to work with them. For this purpose we should create many more exchange schemes, from student to consultant and professorial levels. We should go out of our way to give more than merely a formal welcome to Americans who wish to work here; and we should equip ourselves to make the most of any opportunity of becoming medical ambassadors abroad.

### REFERENCES

Bowers, J. Z. (1963). *Lancet*, 655, i.
Colt, E. (1962). University College Hospital Magazine, **46**, 70.

